# Cholinesterase inhibitors for gait, balance, and fall in Parkinson disease: a meta-analysis

**DOI:** 10.1038/s41531-021-00251-1

**Published:** 2021-11-25

**Authors:** Jia-Hung Chen, Tsai-Wei Huang, Chien-Tai Hong

**Affiliations:** 1 Department of Neurology, Shuang-Ho Hospital, Taipei Medical University, New Taipei, Taiwan; 2 Cochrane Taiwan, Taipei Medical University, Taipei, Taiwan; 3 School of Nursing, College of Nursing, Taipei Medical University, Taipei, Taiwan; 4 Center for Nursing and Healthcare Research in Clinical Practice Application, Wan Fang Hospital, Taipei Medical University, Taipei, Taiwan; 5 Department of Neurology, School of Medicine, College of Medicine, Taipei Medical University, Taipei, Taiwan

**Keywords:** Outcomes research, Parkinson's disease

## Abstract

Gait disturbance and imbalance are the major symptoms of Parkinson disease (PD), with fall being the most undesirable consequence. However, few effective evidence-based treatments are available for alleviating these symptoms and preventing falls. Cholinesterase inhibitors (ChEIs) are a well-established treatment for PD dementia with possible impacts on gait, balance, and fall reduction. The present study involved a meta-analysis of randomized controlled trials (RCTs) to investigate the effects of ChEIs on gait, balance, and fall in patients with PD. We searched for studies using the PubMed, Embase, and Web of Science databases. The major outcomes were effects on gait parameters, balance, and fall. This study was registered with PROSPERO (CRD42021254733). Five RCTs were included in the present meta-analysis. ChEIs did not significantly increase gait speed in PD patients (mean difference [MD]: 0.03 m/s, 95% confidence interval [CI]: −0.02 to 0.07, *p* = 0.29). However, ChEI treatment significantly decreased step or stride variability during the single task (standard MD: −0.43, 95% CI = −0.79 to −0.06, *p* = 0.02). Regarding fall and balance, trending but nonsignificant beneficial effects were observed with ChEI treatment. In conclusion, although ChEI treatment did not significantly improve gait speed and reduce fall, it can significantly reduce step or stride variability. Considering that gait disorder is a challenging issue in patients with PD and that ChEIs are generally tolerable, the present meta-analysis may provide more evidence for the benefit of ChEIs on PD gait disturbance as an alternative treatment consideration.

## Introduction

Parkinson disease (PD) is the second most common neurodegenerative disease^1^. The cardinal motor symptoms of PD include rigidity, tremor, bradykinesia, and gait disturbance. Unlike the first three symptoms, gait disturbance does not respond well to dopaminergic replacement therapy^2^. The postural instability and gait disturbance subtype of PD deteriorated rapidly compared with the other subtypes^3^. Traditional subthalamic deep brain stimulation usually does not alleviate gait disturbance and sometimes may even worsen it^4^. The most devastating consequence of gait disturbance and imbalance is fall, which results in fracture, traumatic brain injury, and fear of walking. Gait disturbance and fall are substantial threats to the quality of life and mortality of individuals with PD^5^.

The risk factors for gait disturbance and falls in PD are variable. It could be age-related, such as anxiety, depression, osteoporosis, polypharmacy, weakness, or due to PD-related symptoms such as rigidity, cognitive impairment, dyskinesia, postural instability, and slow mobility^6^. In people with PD, cognitive dysfunction is a major contributor to gait disturbance and fall, especially the most detrimental form of gait disturbance, that is, freezing of gait (FOG)^7,8^. The signature manifestation of cognitive dysfunction-related FOG is a remarkable worsening of gait during dual-task challenges, which are usually a gait test accompanied by a calculation^9^.

Evidence of cognitive contributions to gait and fall are well-acknowledged, but the precise mechanisms are not completely understood. In those people with dementia, gait impairments are more prevalent than in normal aging and are related to the severity of cognitive impairment^10^. Similar results are also demonstrated in risk of falls that older adults with moderate to the severe cognitive impairment have a higher risk^11^. Attention and executive function are thought to be responsible for postural and gait stability^12,13^. According to the pre-clinical studies, cortical cholinergic neurons-lesioned parkinsonism mice demonstrated great gait disturbance and fall propensity^14^, which is suspected to result from the disruption of cortical processing of task and movement cues for the striatum^15^. In addition, augmentation of cholinergic effect by either rivastigmine or donepezil partially attenuated the symptoms in this dual-lesioned model^16,17^. More than cognitive dysfunction, degeneration of the brainstem cholinergic neurons, pedunculopontine nucleus (PPN), and laterodorsal tegmental nucleus (LDT) are also proposed as one of causative t for gait disturbance in individuals with PD^18^.

Cholinesterase inhibitors (ChEIs) are widely used for the management of dementia, including Alzheimer disease and vascular dementia. Regarding PD dementia, at present, only rivastigmine provides a significant evidence-based therapeutic effect^15^. Numerous studies have examined the effects of ChEIs on patients with PD, but most have focused on cognition and dementia^19–22^. Some pre-clinical studies investigated the impact of gait, balance, and fall and demonstrated possible benefits^16,17^. However, most were small-scale clinical studies, providing low evidence for benefits. The present study aimed to summarize the effect of ChEIs on gait, balance and falls in patients with PD.

## Results

### Summary of the included studies

**Fig. 1:**
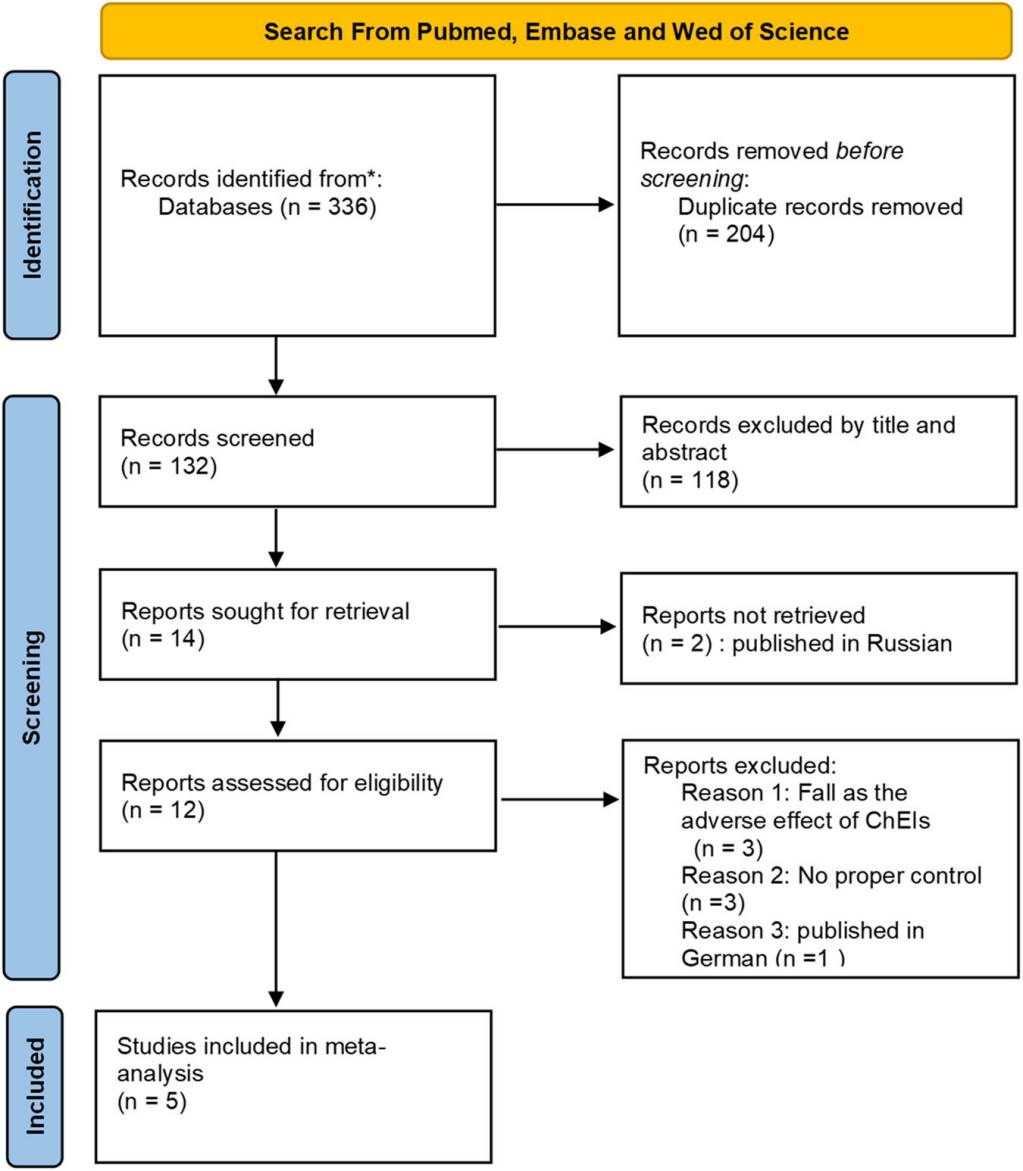
The study selection flowchart. Randomized controlled trials published before May 2021 in the PubMed, Embase, and Web of Science databases were searched following the Preferred Reporting Items for Systematic Review and Meta-Analysis (PRISMA) guidelines.

Figure 1 displays the study selection flowchart. Our initial search yielded 336 studies, 204 of which were eliminated due to duplication. The remaining 132 studies were subjected to title and abstract screening, and 118 were excluded. The final 12 studies, except two that were published in Russian, were entered in the full text review. Three studies were excluded because fall was the adverse effect of ChEI treatment and not the major outcome, and another three studies were excluded due to the lack of a placebo control. One study was excluded as it was published in German. The remaining five eligible RCTs^23–27^ were included in our analysis. These RCTs were published between 2010 and 2020 and had sample sizes ranging from 20 to 130. The mean age of patients was approximately 70 years, and all study cohorts had male preponderance. All five studies recruited patients diagnosed as having idiopathic PD. Two studies specifically enrolled patients with a history of fall. Rivastigmine was prescribed in two studies, and in the remaining three, donepezil was prescribed (Table 1). All of them were categorized into low risk or some concerns regarding the risk of bias (Table 2).

**Table 1:** Characteristics of the included studies.

Author (year)	Inclusion criteria	No. of patients (male, %)	Age (years)^a^	Disease duration (years)^a^	Intervention	Outcome
Chung et al.^23^	Falling or nearly falling ≥2/week	23 (15, 65%) Cross-over design	68.3 ± 10.8	10 ± 5.6	Donepezil: 5 mg for 3 weeks plus 10 mg for 3 weeks	Gait speed ABC scale Fall
Henderson et al.^26^	H&Y stage 2–3, at least one fall in the previous year	E: 65 (35, 54%) C: 65 (46, 71%)	E: 71 (54–90) C: 69 (46–88)	E: 8 (5–13) C: 9 (5–13)	Rivastigmine: 3 mg titrated up to 12 mg/day for 32 weeks	Gait speed and variability, controlled leaning balance score, fall
Li et al.^25^	UK brain bank diagnosis criteria	E: 41 (30, 73%) C: 40 (21, 53%)	E: 67.5 [52.7–71.1] C: 66.9 [53.8–70.3)	E: 5.3 [2.4–7.1] C: 5.5 [2.6–8.0)	Rivastigmine: 6 mg/day for 12 months	Fall
Mancini et al.^24^	H&Y stage 2–4	45 (unknown), Cross-over design	69 ± 7	7 ± 5	Donepezil: 5 mg: 3 weeks plus 10 mg for 3 weeks	Gait speed and variability, ABC scale
Stuart et al.^27^	H&Y stage 2–4	20 (14. 70%), Cross-over design	72.68 ± 7.58	Unknown	Donepezil: 5 mg for 2 weeks	Gait speed and variability

*ABC scale* activities-specific balance confidence scale; *C* control, *E* experimental

^a^Presented as mean ± standard deviation, or mean [95% confidence interval], or median (range).

**Table 2:** Assessment of methodological quality of included trials (RCT evaluated by RoB 2.0).

Author (year)	Bias caused by adequacy of randomization	Bias caused by deviations from intended interventions	Bias caused by missing data of dropouts	Bias in measurement of outcomes	Bias in selection of reported results	Overall risk of bias
Chung et al.^23^	Some concern	Low risk	Low risk	Low risk	Low risk	Some concerns
Handerson et al.^26^	Some concern	Low risk	Low risk	Low risk	Low risk	Some concerns
Li et al.^25^	Some concern	Low risk	Low risk	Low risk	Low risk	Some concerns
Mancini et al.^24^	Low risk	Low risk	Low risk	Low risk	Low risk	Low risk
Stuart et al.^27^	Low risk	Low risk	Low risk	Low risk	Low risk	Low risk

### Effects on gait

**Fig. 2:**
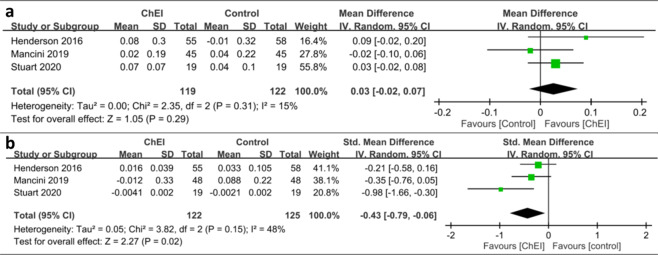
Effect of cholinesterase inhibitors (ChEIs) on the gait speed and step/stride variability of patients with Parkinson disease (PD). **a** ChEIs did not significantly increase gait speed in patients with PD (Mean Difference [MD]: 0.03 m/s, 95% confidence interval [CI]: −0.02 to 0.07, *p* = 0.29). **b** A significant beneficial effect on step or stride variability during the single task (Standardized MD [SMD]: −0.43, 95% CI: −0.79 to −0.06, *p* = 0.02) was demonstrated.

Our meta-analysis of gait parameters revealed that ChEIs did not significantly increase gait speed in patients with PD (MD: 0.03 m/s, 95% CI: −0.02 to 0.07, *p* = 0.29). The heterogeneity of this analysis was nonsignificant (*I*^2^ = 15%; Fig. 2a). By contrast, a significant beneficial effect on step or stride variability during the single task (SMD: −0.43, 95% CI: −0.79 to −0.06, *p* = 0.02) was demonstrated. The heterogeneity of this analysis was nonsignificant, with *I*^2^ being 48% (Fig. 2b).

### Effects on balance and fall

**Fig. 3:**
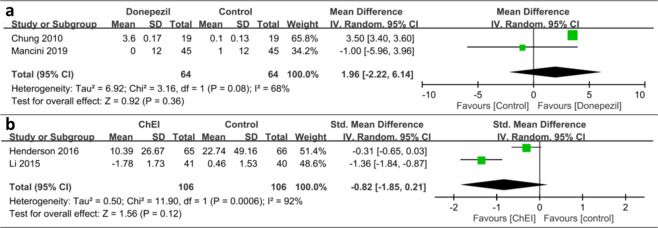
Effect of cholinesterase inhibitors (ChEIs) on the balance and fall frequency of patients with Parkinson disease (PD). **a** Trending but nonsignificant beneficial effects were conferred for balance improvement (Standardized Mean Difference [SMD]: 1.96, 95% Confidence Interval [CI]: −2.22 to 6.14, *p* = 0.36). **b** The effect of ChEIs on fall reduction was not significant (SMD: −0.82, 95% CI: −1.85 to 0.21, *p* = 0.12).

Regarding balance and fall, trending but nonsignificant beneficial effects were conferred. Two studies were added into the quantitative analysis for balance improvement, which revealed highly heterogenous (*I*^2^ = 68%) and nonsignificant results of balance improvement (SMD: 1.96, 95% CI: −2.22 to 6.14, *p* = 0.36; Fig. 3a). Regarding fall reduction, two studies demonstrated highly variable numbers of falls per year, which leads to the high heterogenicity of the effect size (*I*^2^ = 92%). The effect of ChEIs on fall reduction was not significant (SMD: −0.82, 95% CI: −1.85 to 0.21, *p* = 0.12).

## Discussion

We summarized the results of five RCTs published between 2010 and 2020. All studies recruited patients diagnosed as having idiopathic PD by neurologists. The present study demonstrated that although ChEIs did not significantly increase gait speed in individuals with PD, step or stride variability significantly improved. Treatment with ChEIs provided trending improvements in fall prevention and balance, but the results were highly variable. Considering that patients with PD generally tolerated ChEIs, they were a possible treatment alternative for gait disturbance for these patients.

Gait is a complex task involving the cognitive, sensorimotor, and cerebellar systems^28^. The cholinergic transmission is vital in performing this task modulation of cognition and motor systems^18^, since the basal forebrain cholinergic nuclei, the nucleus basalis of Meynert (NBM), and the substantia innominata project to the cerebral cortex, thalamus, and striatum. PD dementia is associated with NBM degeneration^29^, and patients with PD dementia converted from PD-related mild cognitive impairment exhibited more significant loss of the NBM^30^, which may be responsible for worse gait performance. In people with PD, cognitive impairment in PD also limits the execution of complex tasks, such as gait. The severity of PD-FOG, the most devastating gait disturbance in PD, is inversely associated with the set-shifting performance. For those with PD, their dual-task (simultaneous cognitive load) walk test demonstrated low gait speed^31^, increased gait variability^32^, and poor coordination^33^. ChEIs improve cognition in patients with PD dementia through the cholinergic augmentation effect^19^, which may further improve gait disturbance. The cholinergic system is also present in the brainstem PPN and LDT that control the gait performance. A lesion in the PPN impaired motor coordination in experimental animals^34^, and the loss of cholinergic neurons in the PPN was correlated with falls in people with PD^35^. In addition, the thalamus is a projection of the PPN, and the reduction of thalamic cholinergic projection was greater in those with PD-related falls^36^. Central-acting ChEIs enhance brain cholinergic activity, modulating gait through cognitive and motor coordination aspects.

Step or stride variability is a cardinal feature of gait disorders in PD and is distinctly affected by stride length and frequency^37^. People with PD usually lost the ability to regulate the stride-to-stride fluctuations while walking, especially in those with FOG^38^. Disability in stride variability is also considered a risk factor of cognitive decline^39^, and the relationship between step time variability and falls has been identified^40^. The present study demonstrated that ChEIs improved step or stride variability in patients with PD, both of which are highly associated with cognition^41^. This effect was observed in all included studies, especially that conducted by Stuart et al.^27^. On the other hand, only an increasing trending in gait speed was observed upon ChEI treatment. The slowness of gait in PD may result from bradykinesia, FOG, and hesitation. The latter two conditions may be triggered by anxiety or fear of fall, and they were more responsive to anxiolytic agents than ChEIs^42^.

Balance control on movement (dynamic movement) and stillness (static balance) requires integrating all levels of the nervous system. The basal ganglia are involved in controlling balance via the thalamic–cortical–spinal loops and via the brainstem PPN, cholinergic neurons, and the reticulospinal system^43^. For patients with PD, rigidity, bradykinesia, impaired sensory integration, and cognitive impairment contribute to imbalance, and the latter two factors are dopamine insensitive^44^. Poorer balance was associated with the PPN–thalamic cholinergic system^35^, and the PPN deep brain stimulation may confer beneficial effects on balance in patients with PD^45^. The present meta-analysis was on balance from two RCTs^23,24^, and the improvement was only marginal. Another RCT conducted by Handerson et al.^26^ reported a significant benefit of rivastigmine on balance, assessed by controlled leaning balance. However, the authors presented only the categorical outcome of the tests as low, medium, high, and very high, which made a quantitative meta-analysis impossible.

Furthermore, Chung et al.^23^ revealed inconsistent results. However, it demonstrated the relative benefit of donepezil on balance assessed by the Activities of Balance Confidence, but no difference was noted when the Berg Balance Scale was used. Throughout the present meta-analysis, a significant benefit of ChEIs on balance in patients with PD was not demonstrated.

Falls are common among people with PD, and some have recurrent falls^46^. The risk factors of fall in PD can be categorized into generic (i.e., old age, osteoporosis, vertigo, or anxiety) and PD-related aspects (axial rigidity, cognitive impairment, medications, or FOG)^6^. ChEI treatment was speculated to alleviate cognitive impairment and FOG; however, it is not effective against other factors. The present analysis included two studies on treatment with rivastigmine, demonstrating a trending but nonsignificant effect on fall prevention. One of the studies conducted by Li et al.^25^ recruited PD patients with different cognition statuses (normal, MCI, or dementia) without mentioning previous fall history and found a significant reduction in the number of falls compared with the placebo group (1.82 ± 1.99 versus 4.26 ± 1.63 per year, *p* < 0.01). The rivastigmine group also had a better cognitive performance at study completion.

On the other hand, Handerson et al.^26^ recruited PD patients with at least one fall, the strongest fall predictor, in the previous year. At baseline, rivastigmine and placebo groups exhibited nonsignificant differences in the number of falls (approximately five falls in the previous year). At study completion, the rivastigmine group had 1.4 ± 2.47 falls per month (equal to 16.8 ± 29.64 per year) compared with 2.4 ± 4.40 falls per month (equal to 28.8 ± 52.8 per year) in the placebo group. Although a significant difference was noted in the number of falls between groups, both groups showed a remarkable increase in the number of falls compared with baseline. The number of falls was greater than that reported by Li et al.^25^. Because of the highly variable number of falls, the present meta-analysis failed to demonstrate significant fall prevention from the two notable results. In addition, one study included in the meta-analysis, conducted by Chung et al.^23^. investigated the number of falls in the placebo and donepezil groups. They found that fall frequency per day in the placebo group was 0.25 (equal to 91.25 per year) compared with 0.13 in the group (equal to 47.45 per year) taking donepezil (*p* < 0.05). However, that study did not provide information on the baseline number of falls, which limits the utility of its inclusion into a quantitative meta-analysis.

In summary, three studies reported significant fall prevention associated with ChEI treatment in PD patients. However, a considerable difference in the number of falls per year (1.82–91.25) between the three studies rendered it difficult to confirm a significant and homogenous result from the meta-analysis. We argued that ChEIs might prevent falls in PD patients; however, further studies are required to clarify the benefit among a specific group of people with PD.

The strength of the present study was that it analyzed not only fall but also gait parameters and balance. Although not all five studies had complete information, with the help of a meta-analysis, we identified the significant benefit of the ChEIs rivastigmine and donepezil on gait variability and the trending benefit on gait speed and fall reduction. The discrepancy of the response upon ChEI treatment among the gait parameters and falls is noted. The possible explanation is that the gait variability is highly cognition-depended^47^, which indicated greater contribution of cholinergic system. On the other hand, gait speed, balance, and fall are affected by the combination of multiple neurotransmitters systems, and the augmentation of single system cannot result in significant impact^48^. Considering that gait disturbance is challenging in the management of PD, a tolerable and possibly effective treatment involving ChEIs may be considered. Rivastigmine, which is effective for PD dementia management^49^, is the preferred treatment of choice with dual benefits for people with PD. However, the adverse effect of rivastigmine, such as nausea, vomiting, and tremor aggravation^19^, should be cautiously monitored. Further RCTs are warranted to clearly delineate the cognition-based and non-cognition-based therapeutic benefits of ChEIs on PD.

This study had some limitations, and heterogeneity was inevitable. Although rivastigmine and donepezil are both ChEIs, their effects on PD dementia are distinct. Only rivastigmine is effective for PDD, and its inhibition of both butyrylcholinesterase and acetylcholinesterase explains its difference from donepezil, which only inhibits acetylcholinesterase. Merging the results from these two ChEIs introduces heterogeneity. PD patients were also not uniformly selected. Some researchers have specifically recruited PD patients with falls, whereas others have included general PD patients. Besides, although FOG is the most notorious gait disturbance in PD, only one study assessed the effect of ChEI on PD-FOG by the FOG questionnaire, which demonstrated no significant benefit^26^. Finally, to conduct a gait analysis, the recruited PD patients had to be at least able to walk independently, which excluded PD patients with the most severe gait disturbance and highest risk of fall.

In conclusion, ChEI treatment significantly improved gait variability in PD patients and provided trending benefits in terms of gait speed and fall prevention. Considering the tolerability and cognitive benefits, ChEIs may be considered an alternative medical treatment for gait disturbance in PD patients. Future RCTs are warranted to compare the effect between rivastigmine and donepezil and the anatomical origin, either NBM or PPN cholinergic neurons, of gait benefit.

## Methods

### Inclusion criteria

This study included only randomized controlled trials (RCTs) that investigated the effects of ChEIs (including rivastigmine, donepezil, and galantamine) on gait, balance, and fall in patients with idiopathic PD. RCTs were required to clearly report the patient inclusion and exclusion criteria; the process of randomization; method, dosage, and duration of ChEIs; and a comprehensive assessment of gait, balance, and fall. This study was registered with PROSPERO (CRD42021254733).

### Literature search strategy

We searched for RCTs published before May 2021 in the PubMed, Embase, and Web of Science databases following the Preferred Reporting Items for Systematic Review and Meta-Analysis (PRISMA) guidelines. The search keywords are as follows: (“Parkinson’s disease” [title/abstract] OR “Parkinson disease” [title/abstract]) AND (“cholinesterase inhibitors” [title/abstract] OR “donepezil” [title/abstract] OR “rivastigmine” [title/abstract] OR “galantamine” [title/abstract]) AND (“gait” [title/abstract] OR “walk” [title/abstract] OR “balance” [title/abstract] OR “fall” [title/abstract] OR “falls” [title/abstract]). Only studies published in English were included.

### Data extraction

Baseline and outcome data were independently retrieved by two reviewers (J-H.C. and T-W.H.). Furthermore, data on study designs, study population characteristics, and inclusion and exclusion criteria were extracted. Decisions recorded individually by the reviewers were compared, and disagreements were resolved by a third reviewer (C-T.H.).

### Outcomes

The primary outcome was the effect of ChEIs on gait parameters, including speed and variability. The secondary outcome was changes in balance and fall. If more than one assessments on balance scale was used in a single study, the more commonly used scale was selected for outcome analysis.

### Appraisal of methodological quality

Two reviewers (C-T.H. and H-T.H.) independently assessed the methodological quality of each study using the revised risk of bias (version 2.0) method, as recommended by the Cochrane Collaboration. The included studies were scored to determine whether they had a high, medium, or low overall risk of bias. The risk of bias was calculated through the assessment of five domains: bias resulting from the randomization process, bias resulting from deviations from intended interventions, bias resulting from missing outcome data, bias in the measurement of outcomes, and bias in the selection of reported results.

### Statistical analysis

Data were entered and analyzed using Review Manager 5.3 (The Cochrane Collaboration, Oxford, England). A meta-analysis was performed following the PRISMA guidelines. The standard deviation was calculated using the provided confidence interval (CI) limits, standard errors, or interquartile ranges, where appropriate. The effect sizes of continuous outcomes were reported as the standardized mean difference (SMD). The precision of effect sizes was reported using a 95% CI. A pooled estimate of weighted mean difference (WMD) was computed using the DerSimonian and Laird random-effects method. A statistically significant result was indicated by a *p* value of <0.05 or a 95% CI that did not include 1 in the relative risk ratio and 0 in the WMD estimation. Statistical heterogeneity and inconsistency in treatment effects across the studies were evaluated using the Cochrane *Q*-test and *I*^2^ statistic, respectively. Statistical significance was set at a *p* value of <0.10 for the Cochrane *Q*-test. Statistical heterogeneity across the studies was assessed using the *I*^2^ statistic, which quantifies the proportion of total outcome variability across studies.
